# A multicenter epidemiologic analysis of the injuries affecting female and male collegiate basketball players

**DOI:** 10.1002/jeo2.70414

**Published:** 2025-09-04

**Authors:** Rishi Trikha, Nicole J. Hung, Daniel J. Chernoff, Clayton Del Prince, Sharon L. Hame, Kristofer J. Jones, Thomas J. Kremen

**Affiliations:** ^1^ UCLA Department of Orthopaedic Surgery David Geffen School of Medicine at University of California Los Angeles Los Angeles California USA; ^2^ Department of Orthopaedic Surgery University of Missouri, Kansas City Kansas City Missouri USA; ^3^ Department of Orthopaedics University at Buffalo Cheektowaga New York USA

**Keywords:** basketball, collegiate, gender, injuries, return to sport

## Abstract

**Purpose:**

The global rise in popularity of basketball has prompted an increased emphasis on understanding the injury patterns affecting players. This study analysed injury epidemiology and return to sport outcomes in Division I male and female collegiate basketball players. The authors hypothesise that ankle injuries are amongst the most common in this population and that there are similarly comparable injury rates between genders.

**Methods:**

A retrospective review of a de‐identified conference‐specific injury database for male and female Division I collegiate basketball players from 2017 to 2021 was conducted. Injuries were stratified by anatomic location, gender, time missed from practice/competition, and diagnosis. Injury incidence was normalised per 1000 athlete exposure hours (AEH) and relative risk (RR) assessed gender‐based differences.

**Results:**

Of 853 athletes, 663 (77.7%) sustained 4532 injuries. Females were more likely to sustain any injury with injuries per 1000 AEH being 6.27 and 5.55 for males (RR: 1.23, confidence interval [95% CI: 1.15–1.32], *p* < 0.001). The most common injuries were ankle/hindfoot (22.2%), knee (16.5%), head/face (11.7%) and forefoot (9.8%) injuries. Females were significantly more likely to sustain knee (RR: 1.49 [95% CI: 1.27–1.75], *p* < 0.001), head/face (RR: 1.32 [95% CI: 1.09–1.60], *p* = 0.005), or midfoot/forefoot (RR: 1.42 [95% CI: 1.14–1.76], *p* = 0.002) injuries. Females were also significantly more likely to suffer a concussion (RR: 1.75 [95% CI: 1.30–2.36], *p* < 0.001) with 82 of 360 females (22.8%) experiencing a concussion and 64 of 493 males (13.0%). Of all concussions, 12.4% led to absences exceeding 4 weeks.

**Conclusions:**

Female athletes experienced higher overall injury rates and specifically elevated rates of concussive, knee, head/face, and midfoot/forefoot injuries. While lower extremity injuries were most common across both genders, they typically resulted in limited time lost. These findings underscore the need for injury prevention programs addressing multiple body regions and highlight the importance of incorporating gender‐specific considerations into training and return to play protocols to ultimately keep our athletes safe.

**Level of Evidence:**

Level III, retrospective comparative study.

**Abbreviations**
ACL: anterior cruciate ligament
AE: athlete exposures
AEH: athlete exposure hours
CI: confidence interval
IR: injury rate
NCAA: National Collegiate Athletic Association
Pac‐12: Pacific coast conference
RR: relative risk

## INTRODUCTION

With an estimated 450 million female and male basketball players worldwide, there is a growing interest in characterising basketball‐specific injury patterns and their effect on return to sport [13]. Prior studies evaluating gender‐based differences in this area have utilised publicly available data and have largely focused on professional athletes, underscoring the need for investigation of high‐quality injury data [2, 18, 19, 24]. The current study utilises data collected from a variety of healthcare professionals in real‐time in collegiate athletes and hypothesises that ankle injuries are among the most common in this population and that there are similarly comparable injury rates between genders.

Basketball requires a higher frequency of lateral movement and jumping when compared to other sports such as lacrosse, soccer and volleyball [34]. Given the frequent change in direction, rapid acceleration/deceleration, frequent pivoting, repetitive jumping on a hardwood surface, and player‐to‐player contact required in basketball, these athletes are susceptible to unique acute and overuse injuries [19]. While male basketball players have been reported to be at higher risk of sustaining acute contact injuries during competition, female athletes have been reported to have higher rates of sustaining overuse injuries during practice [40]. Furthermore, while the increased incidence of anterior cruciate ligament (ACL) tears in female athletes in all sports has been well‐studied, little is known about other gender‐based injury differences in collegiate basketball players with some studies highlighting the need for further epidemiologic comparative studies [7, 22, 39].

Basketball is one of the most popular sports governed by the National Collegiate Athletic Association (NCAA). During the 2020–2021 season, there were 34,000 NCAA basketball players competing across Divisions I through III [28]. Prior epidemiologic studies demonstrated injury rates ranging from 6.3 to 9.9 injuries per 1000 athlete exposures (AE) for male and 5.9–10.4 injuries per 1000 AEs for female collegiate basketball players [18, 24, 40]. These studies also noted a downtrend in the rate of injuries resulting in missed play over the past three decades up until 2015, possibly due to the implementation of safety measures and rule changes from 2008 to 2015 [6, 26].

Since 2015, additional significant rule changes have been implemented for NCAA women's basketball competition including the transition from two, 20‐min halves to four, 10‐min quarters, extension of the three‐point line to match the distance of the men's line, and the allowance of postposition defenders to place a forearm or hand on the back of the offensive post player [27]. These recent changes along with advances in sports performance technology and medicine highlight the need for contemporary investigations by healthcare professionals of gender‐based injury patterns and return to play parameters following these injuries [19]. By utilising a NCAA Division I conference‐wide injury database, the current study aimed to characterise the types of injuries affecting female and male collegiate basketball players from 2017 to 2021. Upon establishing commonly recognised injury patterns as well as associated return to sport normative data, this study can potentially help guide gender‐specific prevention and treatment protocols. Furthermore, these data can be used to manage return to play expectations in an evidence‐based manner and strengthen the relationship between clinician and athlete.

## METHODS

The deidentified pacific coast conference (Pac‐12) injury database was queried as in prior published works to perform this retrospective cohort study [35, 36]. The query included all male and female collegiate basketball players in the Pac‐12 from 2017 to 2021 who had an ‘injury event’. This ‘injury event’ was our primary outcome and was defined as any injury incurred by the study population either during a practice or a competition in‐season as documented by a member of each institutions medical team which included physicians, athletic trainers or physical therapists. These injuries were then stratified by anatomic location, gender, injury diagnosis and time missed from either practice or competition. Stratifications of anatomic location included head/face, spine/neck, shoulder, elbow/arm, hand/wrist/forearm, chest/abdomen, groin/hip/pelvis/buttocks, thigh, knee, lower leg, ankle and foot. The two most common injuries by anatomic location were further stratified by diagnosis. Time missed was categorised as no time away from sport, between 1 and 4 weeks away from sport and more than 4 weeks away from sport. Repeat injuries were counted as separate injuries if the athlete returned to play and subsequently had to be removed from play.

Percent of athletes affected was defined as the number of athletes who sustained a given injury divided by the total number of athletes included. ‘Estimated athlete risk per year’ was used as a surrogate for risk per athlete‐year since observation periods were not known for each individual athlete due to the data being deidentified. We estimated each athlete's annual risk of injury by dividing the total injuries by the cumulative athlete‐years observed, assuming an average of 2.3‐year career per athlete which assumes an equal number of freshmen, sophomores, juniors, and seniors per year and no athletes transferring/quitting. This allowed for comparison of relative risk (RR) between genders.

To estimate athlete exposure hours (AEH), total season length in weeks was multiplied by 20 h per week as this is the maximum duration of sport‐related activity according to the National Collegiate Athletic Association bylaws, in accordance with prior studies [35, 36]. Team rosters as well as season start and end dates were obtained from publicly available Pac‐12 websites. Publicly available team rosters at each Pac‐12 institution were utilised to determine the total number of collegiate basketball players in the Pac‐12 from 2017 to 2021. This was performed such that athletes who were on the team for multiple years or athletes who transferred institutions within the Pac‐12 conference were not repeatedly counted.

Injury rate (IR) per 1000 AEHs was calculated by the following formula:

IR per1000AEH=[(Number of total in−season injuries)/(Season length in weeks∗20hours/week∗number of athletes on the team)]∗1000.

Microsoft Excel version 16.49 was utilised for all statistical analysis. RR was used to compare estimated athlete risk per year between genders with statistical significance being defined by a 95% confidence interval (CI) not including a value of 1.0 (*p* < 0.05).

## RESULTS

A total of 663 college basketball players out of 853 (77.7%) suffered 4532 total injuries over the study period. Significantly more females suffered an injury (314 out of 360, 87.2%) when compared to males (349 of 493, 70.8%) with the overall injuries per 1000 AEH being 6.27 for females and 5.55 for males (RR: 1.23 [95% CI, 1.15–1.32], *p* < 0.001). The most common injuries between genders were ankle and hindfoot injuries accounting for 1007 out of 4532 (22.2%), knee injuries accounting for 750 injuries (16.5%), head/face injuries accounting for 532 injuries (11.7%), forefoot injuries accounting for 446 injuries (9.8%) and hand/wrist/forearm injuries accounting for 359 injuries (7.9%) (Table 1). Females were significantly more likely to suffer a knee injury (RR: 1.49 [95% CI: 1.27–1.75], *p* < 0.001), a head/face injury (RR: 1.32 [95% CI: 1.09–1.60], *p* = 0.005), or midfoot/forefoot injury (RR: 1.42 [95% CI: 1.14–1.76], *p* = 0.002) (Table 1).

**Table 1:** Musculoskeletal injuries in men's and women's collegiate basketball athletes.

	Women's basketball (360 athletes)				Men's basketball (493 athletes)				RR (95% CI)^a^	*p*‐Value
	Events, *n* (%)	Athletes affected, *n* (%)	Estimated athlete risk per year, %	Injury rate per 1000 AEH	Events, *n* (%)	Athletes affected, *n* (%)	Estimated athlete risk per year, %	Injury rate per 1000 AEH		
Ankle	464 (21.3)	184 (51.1)	23.4	1.3	543 (23.1)	227 (46.0)	24.5	1.3	1.11 (0.97–1.28)	0.18
**Knee**	**393** (**18.0)**	**184** (**51.1)**	**23.4**	**1.1**	**357** (**15.2)**	**169** (**34.3)**	**18.2**	**0.8**	**1.49** (**1.27–1.75)**	**<0.01**
**Head/face**	**246** (**11.3)**	**139** (**38.6)**	**17.6**	**0.7**	**286** (**12.2)**	**144** (**29.2)**	**15.5**	**0.7**	**1.32** (**1.09–1.60)**	**<0.01**
**Foot**	**239** (**11.0)**	**121** (**33.6)**	**15.4**	**0.7**	**207** (**8.8)**	**117** (**23.7)**	**12.6**	**0.5**	**1.42** (**1.14–1.76)**	**<0.01**
Hand/wrist/forearm	177 (8.1)	105 (29.2)	13.3	0.5	182 (7.7)	123 (25.0)	13.3	0.4	1.17 (0.94–1.46)	0.16
Spine/neck	170 (7.8)	91 (25.3)	11.6	0.5	164 (7.0)	107 (21.7)	11.5	0.4	1.16 (0.91–1.49)	0.24
Groin/hip/pelvis/buttocks	137 (6.3)	75 (20.8)	9.5	0.4	213 (9.1)	125 (25.4)	13.5	0.5	0.82 (0.63–1.06)	0.14
Thigh	100 (4.6)	58 (16.1)	7.4	0.3	88 (3.7)	65 (13.2)	7.0	0.2	1.22 (0.88–1.69)	0.23
Shoulder	90 (4.1)	58 (16.1)	7.4	0.3	121 (5.1)	82 (16.6)	8.8	0.3	0.97 (0.71–1.32)	0.86
Lower Leg	88 (4.0)	56 (15.6)	7.1	0.3	99 (4.2)	68 (13.8)	7.3	0.2	1.13 (0.81–1.56)	0.47
Elbow/arm	46 (2.1)	35 (9.7)	4.4	0.1	54 (2.3)	42 (8.5)	4.5	0.1	1.14 (0.74–1.75)	0.56
Chest/abdomen	29 (1.3)	26 (7.2)	3.3	0.1	39 (1.7)	35 (7.1)	3.8	0.1	1.02 (0.62–1.66)	0.94
Total	**2179 (100)**	**314 (87.2)**	**39.9**	**6.3**	**2353 (100)**	**349 (70.8)**	**36.6**	**5.6**	**1.23 (1.15–1.32)**	**<0.01**

*Note*: Bold text represents statistical significance, *p* < 0.05.

Abbreviations: AEH, athlete exposure hours; CI, confidence interval.

^a^ RR, risk ratio, women versus men.

The most common injuries occurred in the ankle with 411 out of 853 athletes (48.2%) being affected. This corresponded with a 23.4% estimated athlete risk per year in females and a 24.5% estimated athlete risk per year in males. Ankle sprains occurred in 336 out of 853 (39.4%) athletes and corresponded with an 18.9% estimated athlete risk per year in females and a 20.2% estimated athlete risk per year in males. Females were significantly more likely to suffer an ankle bony stress injury compared to males (RR: 5.48 [95% CI: 1.17–25.64], *p* = 0.031), although this only occurred in nine female athletes (1.94%) and four male athletes (0.74%) (Table 2).

**Table 2:** Ankle/heel injuries in men's and women's collegiate basketball athletes.

	Women's basketball (360 athletes)			Men's basketball (493 athletes)			RR (95% CI)^a^	*p*‐Value
	Events, *n* (%)	Athletes affected, *n* (%)	Estimated athlete risk per year, %	Events, *n* (%)	Athletes affected, *n* (%)	Estimated athlete risk per year, %		
Sprain	275 (59.3)	149 (41.4)	18.9	385 (70.9)	187 (37.9)	20.2	1.09 (0.92–1.29)	0.32
Strain^b^	68 (14.7)	42 (11.7)	5.3	46 (8.5)	38 (7.7)	4.1	1.51 (0.99–2.30)	0.05
Achilles tendinitis	63 (13.6)	37 (10.3)	4.7	39 (7.2)	33 (6.7)	3.6	1.54 (0.98–2.41)	0.06
Other/not specified	25 (5.4)	22 (6.1)	2.8	36 (6.6)	30 (6.1)	3.2	1.00 (0.59–1.71)	1.00
Impingement/bursitis	16 (3.5)	15 (4.2)	1.9	21 (3.9)	18 (3.7)	1.9	1.14 (0.58–2.23)	0.72
Contusion	8 (1.7)	7 (1.9)	0.9	12 (2.2)	11 (2.2)	1.2	0.87 (0.34–2.23)	0.78
**Stress injury/fracture**	**9** (**1.9)**	**8** (**2.2)**	**1.0**	**4** (**0.7)**	**2** (**0.4)**	**0.2**	**5.48** (**1.17–25.64)**	**0.03**
Total	464 (100)	184 (51.1)	23.4	543 (100)	227 (46.0)	24.5	1.11 (0.97–1.28)	0.14

*Note*: Bold text represents statistical significance, *p* < 0.05.

Abbreviation: CI, confidence interval.

^a^ RR, risk ratio, women versus men.

^b^ Not including Achilles tendon strains.

The second most common injuries occurred in the knee with 353 out of 853 athletes (41.4%) being affected. This corresponded with a 23.4% estimated athlete risk per year in females and an 18.2% estimated athlete risk per year in males. Patellofemoral pain occurred in 157 out of 853 athletes (18.4%) and corresponded with a 10.3% estimated athlete risk per year in females and an 8.19% estimated athlete risk per year in males. Females were significantly more likely to suffer any knee injury (RR: 1.49 [95% CI: 1.27–1.75], *p* < 0.001). After stratifying by diagnosis, females were significantly more likely to suffer patellofemoral pain (RR: 1.36 [95% CI: 1.10–1.93], *p* = 0.008), an ACL injury (RR: 2.88 [95% CI: 1.37–6.03], *p* = 0.005) or a meniscus injury (RR: 2.97 [95% CI: 1.14–7.73], *p* = 0.026) (Table 3).

**Table 3:** Knee injuries in men's and women's collegiate basketball athletes.

	Women's basketball (360 athletes)			Men's basketball (493 athletes)			RR (95% CI)^a^	*p*‐Value
	Events, *n* (%)	Athletes affected, *n* (%)	Estimated athlete risk per year, %	Events, *n* (%)	Athletes affected, *n* (%)	Estimated athlete risk per year, %		
**Patellofemoral pain** b	**103** (**26.2)**	**81** (**22.5)**	**10.3**	**115** (**32.2)**	**76** (**15.4)**	**8.2**	**1.46** (**1.10–1.93)**	**0.01**
Knee contusion	74 (18.8)	48 (13.3)	6.1	71 (19.9)	59 (12.0)	6.4	1.11 (0.78–1.59)	0.58
Knee sprain/strain^c^	55 (14.0)	40 (11.1)	5.1	60 (16.8)	42 (8.5)	4.5	1.30 (0.86–1.97)	0.22
**Other/not specified**	**50** (**12.7)**	**42** (**11.7)**	**5.3**	**35** (**9.8)**	**26** (**5.3)**	**2.8**	**2.21** (**1.38–3.54)**	**<0.01**
Impingment/synovitis/bursitis	28 (7.1)	23 (6.4)	2.9	21 (5.9)	19 (3.9)	2.1	1.66 (0.92–3.00)	0.09
**ACL injuries**	**23** (**5.9)**	**21** (**5.8)**	**2.7**	**10** (**2.8)**	**10** (**2.0)**	**1.1**	**2.88** (**1.37–6.03)**	**0.01**
Osteochondral injuries	17 (4.3)	17 (4.7)	2.2	14 (3.9)	12 (2.4)	1.3	1.94 (0.94–4.01)	0.07
**Meniscus injuries**	**13** (**3.3)**	**13** (**3.6)**	**1.7**	**6** (**1.7)**	**6** (**1.2)**	**0.7**	**2.97** (**1.14–7.73)**	**0.03**
MCL injuries	12 (3.1)	12 (3.3)	1.5	14 (3.9)	14 (2.8)	1.5	1.17 (0.55–2.51)	0.70
IT band syndrome	9 (2.3)	8 (2.2)	1.0	6 (1.7)	6 (1.2)	0.7	1.83 (0.64–5.22)	0.26
Stress reaction/fracture	5 (1.3)	4 (1.1)	0.5	2 (0.6)	2 (0.4)	0.2	2.74 (0.50–14.87)	0.25
Patellar dislocation	3 (0.8)	3 (0.8)	0.4	3 (0.8)	3 (0.6)	0.3	1.37 (0.28–6.75)	0.71
Fracture	1 (0.3)	1 (0.3)	0.1	0 (0)	0 (0)	0.0		NA
**Total**	**393 (100)**	**184 (51.11)**	**23.4**	**357 (100)**	**169 (34.3)**	**18.2**	**1.49 (1.27–1.75)**	**<0.01**

*Note*: Bold text represents statistical significance, *p* < 0.05.

Abbreviations: ACL, anterior cruciate ligament; CI, confidence interval. MCL, medial collateral ligament.

^a^ RR, risk ratio, women versus men.

^b^ Including patellar and quadriceps tendinitis.

^c^ Excluding ACL or MCL injuries.

Although ankle sprains were the most common injury, 259 ankle sprains out of 660 (39.2%) did not result in any time away from sport. Furthermore, although patellofemoral pain was the most common knee injury, 147 out of 218 (67.4%) of these injuries did not result in missed time. Interestingly, 15 out of 115 male athletes (13.0%) and five out of 103 female athletes (4.85%) suffering patellofemoral pain missed greater than 4 weeks away from sport. One hundred and ninety‐one concussions affected 146 out of 853 athletes (17.1%). Females were significantly more likely to suffer a concussion (RR: 1.23, [95% CI: 1.15–1.32], *p* < 0.001). Eighty‐two out of 360 female athletes (22.8%) suffered a concussion as compared to 64 out of 493 male athletes (13.0%). Return to sport data was not specified for 21 out of 191 concussions. Of the remaining 169 concussions, 103 (61.0%) resulted in 1–4 weeks away from sport and 21 (12.4%, 15 women and 6 men) resulted in more than 4 weeks away from sport (Table 4).

**Table 4:** Return to sport data for common injuries in men's and women's collegiate basketball.

Injury	Did not miss time, *n* (%)	Missed time: < 1 week, *n* (%)	Missed time: 1–4 weeks, *n* (%)	Missed time: >4 weeks, *n* (%)	Not specified, *n* (%)	Total, *n*
Women's Basketball						
Ankle sprain	110 (40.0)	45 (16.4)	65 (23.6)	49 (17.8)	6 (2.18)	275
Concussion	0 (0)	18 (16.8)	59 (55.1)	15 (14.0)	15 (14.0)	107
Patellofemoral pain	76 (73.8)	15 (14.6)	6 (5.9)	5 (4.9)	1 (1.0)	103
Hand/wrist sprain	68 (70.1)	15 (15.5)	2 (2.1)	8 (8.2)	4 (4.1)	97
Hip/groin strain	39 (68.4)	10 (17.5)	5 (8.8)	2 (3.5)	1 (1.8)	57
Men's Basketball						
Ankle sprain	149 (38.7)	89 (23.1)	80 (20.8)	61 (15.8)	6 (1.6)	385
Concussion	0 (0)	28 (33.3)	44 (52.4)	6 (7.1)	6 (7.1)	84
Patellofemoral pain	71 (61.7)	22 (19.1)	5 (4.4)	15 (13.0)	2 (1.7)	115
Hand/wrist sprain	60 (56.1)	15 (14.0)	22 (20.6)	9 (8.4)	1 (0.9)	107
Hip/groin strain	55 (50.0)	22 (20.0)	17 (15.5)	11 (10.0)	5 (4.6)	110

## DISCUSSION

This study utilises a multi‐institutional database of NCAA Division I athletes to demonstrate that ankle injuries were amongst the most common in all basketball players and that females suffered relatively higher rates of concussions, knee injuries, head/face injuries and foot injuries when compared to males. There is a relative paucity of research on injuries in NCAA basketball players and existing studies often lack gender‐based comparisons or substantial detail [18, 24, 40]. Furthermore, literature evaluating gender‐based differences frequently focuses on professional athletes [2, 19]. To develop effective injury prevention strategies, it is essential to understand the epidemiology of injuries in both female and male basketball players. Accordingly, the current study specifically investigates the injury patterns and gender‐based differences in collegiate basketball players by utilising a relatively large sample size. Despite the current study analysing basketball players from the Pac‐12 conference, the multicentre nature of this data capture strengthens the external validity and the authors believe these results are likely applicable to most basketball players.

The current study demonstrated that ankle injuries were the most common injury, impacting approximately half of all athletes. Among all ankle injuries, sprains were the most common. This is consistent with literature, as ankle sprains have previously been found to be the most common basketball injury at any level of competition [3, 11, 21, 37]. This is attributable to the nature of the sport, as basketball involves repetitive jumping/landing, quick directional changes, rapid stops and contact with other players [20, 37]. In the present study, 56.4% of ankle sprains in females and 61.8% of ankle sprains in males resulted in either no missed time or less than 1 week of missed time. This is slightly lower than previously reported data, which found time loss of less than seven days in 63% of female and 65% of male injuries [37]. Although often perceived as minor, 17.8% of ankle sprains in females and 15.8% of ankle sprains in males resulted in greater than 4 weeks of missed time. One possible rationale for the extended time away from sport is that these ankle sprains are often high energy and, given the functional demands of basketball, could potentially lead to chronic ankle instability [20]. Conversely, 259 ankle sprains out of 660 (39.2%) did not result in any time away from sport indicating that many sprains are low grade with little to no clinical significance.

This study also demonstrated that females were slightly more likely to suffer any injury as compared to males. This finding is in contrast to one of the only prior studies investigating gender‐based differences in injuries in collegiate basketball where men had higher overall injury rates than women [40]. Although this study is not equipped to comment on exact etiologies contributing to our observed difference among male compared to female basketball players possible contributing factors include differences in anatomy (e.g., increased genu valgum in females), joint laxity, training techniques, and jumping/landing characteristics [12]. Psychosocial factors have also been reported to contribute to males under‐reporting symptoms when compared to females [15].

Females were at a significantly higher risk of suffering an ankle bone stress injury/fracture. Female athletes have been reported to be at a higher risk for stress fractures, with oligo/amenorrhoea, biomechanics and low vitamin D intake being implicated as potential risk factors [1]. Although this trend is well‐documented in female runners, the current study indicates that female basketball players are similarly at an elevated risk [1, 8]. Rizzone et al. reported a rate ratio of 1.69 (95% CI: 1.26–2.28) for stress injuries in female versus male collegiate basketball players [32]. Though not directly comparable, the RR observed in the current study was even higher, indicating that clinicians should maintain a high index of suspicion for these injuries in female athletes and implement appropriate return‐to‐play protocols when necessary.

Knee injuries were also more common in female athletes. When looking at individual diagnoses, females had higher rates of patellofemoral pain, ACL injuries, and meniscal injuries. In particular, females had much higher rates of ACL injuries and meniscal injuries, which likely drives the increased rates of female knee injuries and further aligns with current literature describing ACL vulnerability. A systematic review of basketball players at all levels of play demonstrated that the female‐to‐male incidence rate ratio for ACL injuries was 3.33 (CI: 3.10–3.57) [33]. Although not directly comparable, the current study found the relative risk of ACL injuries in females compared to males to be 2.88 (95% CI: 1.37–6.03, *p* = 0.005). While the current study is not equipped to comment on the aetiology of any observed gender disparity in ACL injuries, prior literature has shown that it is likely multifactorial in nature. Due to a mixture of anatomic factors and muscle activation imbalances, female athletes have been reported to land with their knees in dynamic valgus more often than males which may predispose ACL tissues to higher physiologic loads [10]. Anatomic factors that may contribute to females being more at risk for ACL injuries include intercondylar notch size, increased posterior tibial slope, and increased *Q*‐angle [4, 14]. Myer et al. demonstrated that females utilise increased quadriceps activation relative to their hamstrings during sport which potentially decreases dynamic joint stabilisation, thus indicating that neuromuscular factors may also contribute to our observed gender disparity [25]. Hormonal factors may also contribute to ACL injuries among females with oestrogen being shown to inhibit collagen formation by ACL fibroblasts and thus potentially reducing the load‐bearing capacity of the ACL [10]. Engebretsen et al. further highlighted the need for epidemiologic research on gender differences in ACL when presenting data demonstrating that females were more at risk of ACL injuries in pivoting sports [7]. While a majority of the literature and media focuses on ACL injuries in female soccer players, the current findings emphasise that female basketball players are also at significantly elevated risk, underscoring the importance for targeted neuromuscular and other training preventative efforts in this population [5, 9].

Concussions represent another area of concern in basketball, with increased attention being paid to their diagnosis, long‐term health effects and management [29]. These injuries can result from contact with another player, the ball, or with the court during a fall [17]. This study found that 17.1% of all collegiate basketball players sustained a concussion. Females were found to be more at risk for sustaining a concussion. In this series, all players that sustained a concussion missed some time away from sport. Current NCAA rules require immediate removal from play and a stepwise return to sport plan for athletes diagnosed with a concussion, therefore it is expected that all athletes missed some time away from sport [23]. While return to play protocols for concussion are standardised, our findings suggest heightened vigilance for female player and highlights the need for the investigation of gender‐specific factors that may contribute to concussion prevention. The vast majority of athletes were able to return to sport relatively quickly, with only 14.0% of women and 7.14% of men missing more than 4 weeks. Basketball is a high‐risk sport for suffering a concussion and is characterised as a medium/high contact sport [38]. The current study certainly supports this notion and exemplifies the importance of heightened diagnostic suspicion as well as appropriate return to play protocols for both male and female basketball players.

This study has several limitations. Injury diagnoses in the Pac‐12 database can be entered by various allied healthcare team members including athletic trainers, physical therapists, primary care physicians, orthopaedic surgeons, and/or psychologists, introducing potential variability in diagnostic criteria [35, 36]. This methodology also potentially underestimates overuse injuries, minor injuries and/or exacerbation of preexisting injuries that were not reported or did not receive medical attention. Although reporting on AEH is a strength of this study, exposure time was assumed to be the maximum time allotted by the NCAA, which indicates that exposure hours were estimated and not individually tracked. Injuries during practice versus competition were not able to be discerned to maintain privacy of data. Furthermore, an athlete's educational class (freshman, sophomore, etc.) and institutional affiliation were omitted to maintain de‐identification of the data set. This dataset also did not distinguish the reason for an athlete not returning to sport, thus it is possible that factors other than the injury influenced an athlete's return to sport status. Additionally, this dataset lacks diagnostic detail that may allow for further injury risk stratification. For instance, it is unclear whether a diagnosis of an ankle sprain referred to a high or low ankle sprain which would affect return to play time. This study also did not capture information on injury prevention strategies or treatment protocols, precluding analysis of those variables. The study time period also includes the coronavirus pandemic, where the injury incidence in collegiate basketball prepandemic has shown to be different than postpandemic [16]. One methodologic limitation was that this study did not adjust for multiple comparisons; therefore, borderline significant findings should be approached with caution, though in our dataset most gender differences were strongly significant.

## CONCLUSION

This multicentre study provides robust injury data in numerous athletes that enables meaningful gender comparisons across a range of injury types, thereby expanding our epidemiologic understanding. The findings from this study highlight a significantly higher risk of injury in female basketball players and underscore the need for injury prevention programs that address multiple body regions, particularly those associated with prolonged return to play times. These data can be used to tailor neuromuscular training and counsel athletes on expected recovery times. This expectation management can improve communication between healthcare teams and athletes, furthering the push to implement evidence‐based medicine into clinical practice [31]. Future research should build on this study by evaluating specific prevention programs, an area where female‐focused interventions have shown success in other sports [30]. Ultimately, the insights gained from this study can support efforts to reduce injury risk and enhance safety in both male and female collegiate basketball players.

## AUTHOR CONTRIBUTIONS

All authors contributed to the study conception, methodology and design. Data acquisition from the database was obtained and analysed by Rishi Trikha, Daniel Chernoff, Clayton Del Prince and Thomas Kremen. The first draft of the manuscript was written by Rishi Trikha, Daniel Chernoff, Sharon Hame, Kristofer Jones and Thomas Kremen. Further editing was carried out by all editing. Funding acquisition and resources were not necessary for this work.

## CONFLICT OF INTEREST STATEMENT

The authors declare no conflicts of interest.

## ETHICS STATEMENT

IRB NA given the de‐identified nature of the database.

## Data Availability

Encourages data sharing; data available on request from authors.
